# Implications and Limitations of Social Distancing Strategies (SDS) to Mitigate the Impact of the COVID-19 Pandemic

**DOI:** 10.1017/dmp.2020.500

**Published:** 2020-12-22

**Authors:** Krzysztof Goniewicz, Frederick M. Burkle, Amir Khorram-Manesh

**Affiliations:** 1 Department of Aviation Security, Military University of Aviation, Dęblin, Poland; 2 Harvard Humanitarian Initiative, T.H. Chan School of Public Health, Harvard University, Boston, MA; 3 Institute of Clinical Sciences, Department of Surgery, Sahlgrenska Academy, Gothenburg University, Gothenburg, Sweden; 4 Department of Development and Research, Armed Forces Center for Defense Medicine, Gothenburg, Västra Frölunda, Sweden

**Keywords:** Covid-19, panedemic, SDS

The World Health Organization defines a disease outbreak as “the occurrence of diseases in excess of normal expectancy. The number of cases varies according to the size, type, and previous exposure to disease-causing agent.”^1^

Several viral infections have evolved as epidemic/pandemic, transmitted through person-to-person contact, animal-to-person contact, or from the environment or other media. The vulnerable populations are more likely to develop severe illness, and since initially there are no specific treatments or vaccine, the best way to prevent and slow down transmission is information and prevention.^2^

The focus on prevention concerns 2 distinct populations: Health care workers and the general population. The former should use contact and airborne precautions to include personal protective equipment, and the latter should follow public health recommendations and strategies.^3^

It is easier to develop models and measures on the effectiveness of non-pharmaceutical interventions (NPIs), but more complicated to determine when to lift such measures. The data concerning social distancing strategies (SDS) effectiveness and their starting and ending times are limited. A 2007 Proceedings of the National Academy of Sciences of the USA study found that cities that deployed multiple interventions at an early phase of the pandemic had significantly lower death rates. Some early studies show different experience of peak coronavirus rates for 2 Chinese cities during the severe acute respiratory syndrome (SARS) epidemic.^4^ The city, which implemented disease control measures early into the outbreak, had significantly lower numbers of hospitalizations from coronavirus disease (COVID-19) on its peak day than the city, which put measures in place a month into the outbreak.^4^

Studies from the pandemic influenza support the combined use of pharmaceutical and NPIs, emphasizing that, “combination strategies delayed spread, reduced overall number of cases, and delayed and reduced peak attack rate more than individual strategies.”^2^

Several factors can influence SDS. One factor is the trustworthy relationship between politicians and their citizens. Party politics create distrust and worsen this relationship, which is necessary for the implementation of scientific and population-based recommendations during an emerging infectious disease outbreak.^1^ In addition, civilian engagement is necessary to administer hygienic measures and public health recommendations to prevent the spread of the infectious cycle. These factors, in turn, are influenced by other factors such as cultural background, state of poverty or well-being, education, and a functioning infrastructure.^2-4^

SDS have a positive effect for societies on limiting the spread of disease. However, there are some important factors to consider when triggering such measures, such as the initiation and termination time, the level of aggressiveness in implementation of the initial steps, the ways of up- and down-scaling the measures, and the susceptibility of the affected community. While it may be impossible to determine best practices for SDS, there is little doubt that the global COVID-19 experience will lend much evidence to the body of knowledge and the determination of best practices going forward. The result might be early and complete initiation of strategies with no hesitations, and a flexible and educated community, able to handle the basic public health recommendations.^5^
